# Acute Thermotherapy Prevents Impairments in Cutaneous Microvascular Function Induced by a High Fat Meal

**DOI:** 10.1155/2016/1902325

**Published:** 2016-08-09

**Authors:** Jennifer C. Harvey, Bruno T. Roseguini, Benjamin M. Goerger, Elizabeth A. Fallon, Brett J. Wong

**Affiliations:** ^1^Department of Kinesiology & Health, Georgia State University, Atlanta, GA 30302-3975, USA; ^2^Department of Health & Kinesiology, Purdue University, West Lafayette, IN 47907, USA

## Abstract

We tested the hypothesis that a high fat meal (HFM) would impair cutaneous vasodilation, while thermotherapy (TT) would reverse the detrimental effects. Eight participants were instrumented with skin heaters and laser-Doppler (LD) probes and tested in three trials: control, HFM, and HFM + TT. Participants wore a water-perfused suit perfused with 33°C (control and HFM) or 50°C (HFM + TT) water. Participants consumed 1 g fat/kg body weight. Blood samples were taken at baseline and two hours post-HFM. Blood pressure was measured every 5–10 minutes. Microvascular function was assessed via skin local heating from 33°C to 39°C two hours after HFM. Cutaneous vascular conductance (CVC) was calculated and normalized to maximal vasodilation (%CVC_max_). HFM had no effect on initial peak (48 ± 4 %CVC_max_) compared to control (49 ± 4 %CVC_max_) but attenuated the plateau (51 ± 4 %CVC_max_) compared to control (63 ± 4 %CVC_max_,* P* < 0.001). Initial peak was augmented in HFM + TT (66 ± 4 %CVC_max_) compared to control and HFM (*P* < 0.05), while plateau (73 ± 3 % CVC_max_) was augmented only compared to the HFM trial (*P* < 0.001). These data suggest that HFM negatively affects cutaneous vasodilation but can be minimized by TT.

## 1. Introduction

Microvascular dysfunction, broadly defined as a state of enhanced vasoconstriction and reduced vasodilation, has been shown to play a key role in the progression of cardiometabolic-related diseases and thus it acts as an important contributor to chronic diseases such as diabetes, hypertension, and dyslipidemia [1–7]. Hypertriglyceridemia has been established as a risk factor of cardiovascular disease and it has been proposed that microvascular dysfunction precedes macrovascular dysfunction, thus making it important to understand how high fat meals/diets may affect human microvascular function [8–13].

The consumption of a high fat diet can transform acute postprandial microvascular endothelial dysfunction into a chronic vascular disease. High fat meal induced inflammation causes triglyceride-rich lipoproteins to accumulate in the blood. Chronic oxidative stress and the resulting chronic inflammation leads to a prolonged state of elevated circulating inflammatory markers furthering the risk for atherosclerosis and cardiovascular disease [14–17]. Nitric oxide (NO) is a vital molecule for cardiovascular health and vascular function as it regulates platelet aggregation and vascular tone [18–21]. Oxidative stress following a high fat meal may inhibit endothelial NO synthase (eNOS) phosphorylation, an important precursor for NO production in the peripheral vasculature [18, 22]. In addition, antioxidant therapy has been shown to prevent the impairment in endothelial function, which further supports a role for oxidative stress [23–25]. Hypertriglyceridemia and the associated oxidative stress lead to the development of prediabetic peripheral neuropathy, particularly in the sensory afferent nerves [26]. High fat meals can thus negatively affect not only NO-dependent vasodilation but also sensory nerve function. Identifying physiologic systems and nonpharmacological interventions to stunt or prevent these effects are deemed important.

The microvasculature is responsible for the bulk of glucose uptake and regulation of vascular resistance and is often the site of dysfunction that leads to diabetic neuropathy, all of which further underscores the importance of understanding how high fat meals affect microvascular function. A high fat meal can have an acute adverse effect, such as a reduction in whole limb blood flow as well as impaired pulmonary function; however, to our knowledge, there have been no studies directly investigating the effect of a high fat meal on human microvascular sensory nerve and endothelial function [27–30]. The cutaneous vasodilation in response to local skin heating (cutaneous thermal hyperemia) is a reproducible biphasic response: an initial, rapid vasodilation mediated primarily by sensory nerves that is followed by a prolonged plateau mediated largely (~80%) by NO derived from eNOS [31–41]. The cutaneous thermal hyperemic response can therefore be used to noninvasively assess microvascular sensory nerve function and endothelial NO-dependent vasodilation simultaneously [31, 32, 34–41].

Thermotherapy (TT) may have the potential to reduce the negative effects of a high fat meal on microvascular endothelial function and may ultimately provide a way to curb the development of atherosclerosis in a nonpharmaceutical manner by upregulating eNOS and inducing vasodilation of systemic arteries [42–45]. Elevated cardiac output concomitantly increases peripheral blood flow and, presumably, the amount of shear stress necessary to evoke NO production within the vessel [46]. Thus, sauna therapy appears to have remedial effects on microvascular endothelial function and in turn may act as a therapeutic modality for halting atherosclerotic processes [46].

The purpose of this study was to investigate the effect of a high fat meal on both microvascular sensory nerve function and microvascular endothelial function. A secondary purpose was to determine whether exposure to TT prior to a high fat meal could mitigate the detrimental effects of the high fat meal on microvascular function. Specifically, we tested two hypotheses: (1) a high fat meal will blunt both cutaneous microvascular sensory nerve- and endothelial-mediated vasodilation and (2) TT application will reverse the detrimental effects of a high fat meal.

## 2. Methods

### 2.1. Ethical Approval

The Institutional Review Board at Georgia State University approved all protocols used in this study (number H14621). Written informed consent was reviewed and signed by all participants prior to participation in this study. All protocols were performed in accordance with the Declaration of Helsinki.

### 2.2. Participant Recruitment and Screening

Eight participants (4 males and 4 females; aged 18–32 years; BMI 22.8 ± 0.3) were recruited and screened for health history prior to study participation. Vascular and oxidative stress responses to a high fat meal are affected by race/ethnicity. For example, studies have shown a greater detriment in vascular function in Asians compared to Caucasians following an acute high fat meal [23–25, 47–49]. Participants in the present study were Caucasian (*n* = 6) and African American (*n* = 2). None of the participants had any cardiovascular or metabolic disease, skin allergy/disease, and history of adverse reaction to heat stress, were smokers, or took any medication for corresponding conditions. For female participants, phase of menstrual cycle or oral contraceptive use was noted but not controlled for in these studies. Participants reported to the laboratory for a completion of three protocols: control, high fat meal (HFM), and high fat meal plus TT application (HFM-TT). The control protocol (no HFM and no TT) was performed first for each participant. The order of the two HFM protocols was randomized across participants. At least, one day, and no more than seven days, was allowed between the control protocol and the first HFM protocol. At least, seven days, and no more than 10 days, were allowed between the two HFM protocols. All experiments took place in a thermoneutral laboratory (20°C–22°C and 52–56% relative humidity).

### 2.3. Participant Instrumentation

For all protocols, a baseline fasting blood sample (at least eight hours fasted, water ad libitum) via finger stick was analyzed for triglycerides and glucose (Cholestech LDX, Alere; San Diego, CA, USA). Each participant wore a water-perfused suit to either clamp or raise core temperature (see details below). The water-perfused suit covered the entire body except head, hands, feet, and the experimental forearm. A core temperature pill (CorTemp, HQ Inc.; Palmetto, FL, USA) ingested two hours prior to laboratory testing was used to monitor core temperature, which was recorded every 3–5 minutes for the duration of the protocol. Each participant was equipped with a 3-lead electrocardiogram and a blood pressure cuff was placed on the right arm. Blood pressure was measured via automated brachial oscillation every 5–10 minutes (Connex 6300, Welch-Allyn; Skaneateles Falls, NY, USA) for the duration of the protocol. An index of skin blood flow was measured at two sites on the dorsal aspect of the left forearm via laser-Doppler flowmetry and each laser-Doppler probe was housed within a local skin-heating unit that was used to control local skin temperature (moorVMS HEAT and LDF2, Moor Instruments; Devon, UK). Each local heater and laser-Doppler probe was affixed to the skin with double-sided adhesive discs (Electrode Washers E432, In Vivo Metric; Healdsburg, CA, USA). Participants were seated in a recliner in the semirecumbent position with both arms at heart level for all protocols.

### 2.4. Local Heating Protocol

Both skin heaters were increased from 33°C to 39°C at a rate of 0.1°C/second to evoke submaximal vasodilation following a 10 min baseline [34]. This rate and magnitude of local heating have been shown to induce vasodilation that is ~80% NO-dependent [36–39]. Once a plateau in skin blood flow was achieved (approximately 30 minutes of heating), the skin heaters were increased to 43°C  to elicit maximal vasodilation [31, 32, 34, 40, 41, 50]. This heating protocol allows for assessment of both sensory nerve function and NO-dependent microvascular endothelial vasodilation [11, 34, 39, 51].

### 2.5. High Fat Meal (HFM)

The high fat meal used in this study (ice cream) was based on two previous studies demonstrating that 1 g fat per kilogram body mass delivered via ingestion of ice cream attenuates pulmonary function and increases exhaled NO, a marker of pulmonary inflammation [27, 30]. Fat consumption for each participant was calculated as one gram of fat per kilogram of body weight (serving size: 88 g, total fat: 9 g; [27, 30]). Servings were measured via electronic balance scale (Ohaus, Scout Pro; Parsippany, NJ, USA) to the nearest one-hundredth of a gram. Participants consumed on average 672.43 ± 53.70 g of ice cream. Each serving of ice cream provided 6 g saturated fat, 35 mg cholesterol, 20 g carbohydrate, and 4 g protein. Participants were allotted 20 minutes to consume the ice cream.

### 2.6. Experimental Protocol 1: Control (No HFM, Thermoneutral)

Following a baseline blood sample, participants underwent an initial local heating protocol as described above in order to assess their control sensory nerve function and NO-dependent vasodilation in the absence of a HFM. The water-perfused suit was perfused with thermoneutral water (33-34°C; Sahara S21 Stainless-Steel Heated Bath Circulator, ThermoFisher Scientific; Waltham, MA USA) to clamp core temperature.

### 2.7. Experimental Protocol 2: High Fat Meal (Thermoneutral)

**Figure 1:**
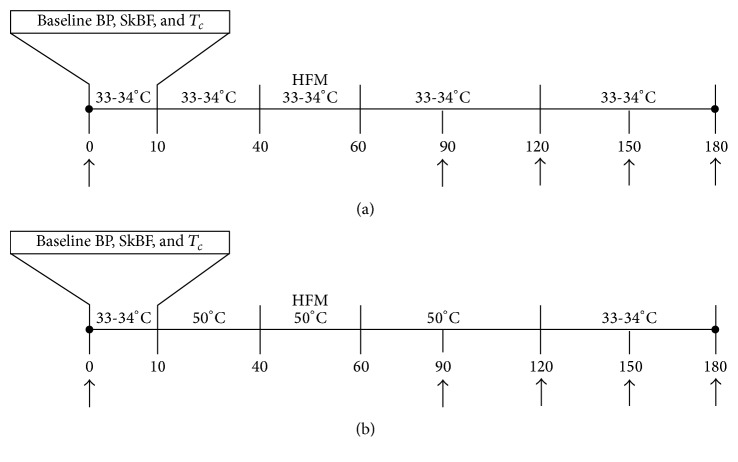
Schematic illustration of the experimental protocol. (a) HFM (thermoneutral) protocol and (b) HFM-TT protocol. Arrows indicate time points where finger stick blood samples were taken. Temperatures of 33-34°C (thermoneutral) and 50°C (thermotherapy) indicate the temperature of the water pumped through water-perfused suit. BP: blood pressure; SkBF: skin blood flow; *T*_*c*_: core temperature; HFM: high fat meal.

The experimental protocol is shown in Figure 1(a). Participants reported to the laboratory and underwent a fasting blood sample and rested for 10 min in the semirecumbent position, during which baseline data was acquired. Next, the water-perfused suit was connected to the water pump and perfused with water at 33°C for 30 minutes to clamp core temperature. After 30 minutes, participants consumed the high fat meal within the 20 minutes allotted. Upon completion of the high fat meal, participants underwent an additional two hours of exposure to thermoneutral water. At the end of the control intervention, a second finger stick blood sample was taken. Previous studies have shown endothelial dysfunction to occur two hours after a high fat meal [1–3].

### 2.8. Experimental Protocol 3: High Fat Meal + Thermotherapy

The experimental protocol is shown in Figure 1(b). This protocol was the same as protocol two (above) with the exception that participants underwent TT application instead of a control intervention. Thermotherapy was applied by pumping 50°C water through the water-perfused suit for 110 minutes: 30 minutes prior to the high fat meal, during HFM consumption (20 min), and 60 minutes after high fat meal. The goal of this protocol was to increase core temperature ~0.2–0.3°C above baseline and also increase skin blood flow while avoiding pronounced vasodilation that could affect the subsequent local heating response (i.e., a ceiling effect due to elevated skin blood flow). Extensive pilot work was performed to determine that this duration of heating was sufficient to increase skin blood flow above baseline and increase core temperature ~0.2–0.3°C above baseline core temperature. At the end of the 90 min of TT application, the temperature of the water was reduced to 33-34°C to allow the participant's core temperature and skin blood flow return to thermoneutral levels. Blood samples were taken thirty minutes after completing the high fat meal and every 30 minutes thereafter until the end of the protocol.

### 2.9. Data Analysis

All data were sampled at 100 Hz (PowerLab 16/35, ADInstruments; Colorado Springs, CO, USA) and stored to the hard drive of a laboratory computer (iMac, Apple; Cupertino, California, USA). Cutaneous vascular conductance (CVC) was calculated as laser-Doppler flux divided by mean arterial pressure and was normalized as a percentage of maximal vasodilation (%CVC_max_). There were no observable or statistical differences in %CVC_max_ between the two laser-Doppler sites within subjects for a given trial so values from each site were averaged.

Baseline values across trials and at various time points within a trial were compared to ensure that changes in %CVC_max_ were not due to baseline shifts in response to either the high fat meal or the mild heat stress. For the control trial, the pre-local heating baseline was averaged over the three-minute period immediately preceding the local heating protocol. For both the HFM and HFM-TT trial, three different time points were analyzed: (1) pre-high fat meal baseline was taken as the three-minute period immediately preceding initiation of either the thermoneutral period for the HFM trial or mild heat stress for the HFM-TT trial; (2) post-high fat meal baseline was taken as the three-minute period immediately preceding the end of the thermoneutral period or the mild heat stress; and (3) pre-local heating baseline was taken as the three-minute period immediately preceding the beginning of the local heating protocol.

For the local heating responses, %CVC_max_ data were analyzed as follows. The initial peak is a rapid and transient response; data were thus averaged over a 30–60-second period corresponding to the highest values prior to a decrease in %CVC_max_ (onset of the nadir) of the local heating response [33–35, 40, 41]. Data for the plateau were averaged over a stable 3–5-minute period when the local heaters were held at 39°C  [31, 33, 34, 40, 41]. For maximal CVC, data were averaged over a stable three-minute period when the local heaters were at 43°C.

Blood samples were analyzed for differences between baseline during the control, HFM, and HFM-TT trials. The baseline values were compared to the values two hours after the high fat meal in the HFM and HFM-TT trials.

All data were analyzed via SPSS 22 (IBM Corporation; Armonk, NY, USA) and presented as mean ± SEM. Statistical significance was initially set at *α* = 0.05, with Bonferroni corrections when appropriate. A Shapiro-Wilk test was performed to determine if the data were normally distributed. All data were normally distributed; however, only *P* values for blood triglycerides are reported because of the large variability of the blood triglyceride data. One-way ANOVAs were used to examine differences in (a) baseline levels of TRG and GLU across experimental conditions, (b) baseline systolic, diastolic, and mean arterial blood pressure across experimental conditions, (c) changes in HR across conditions, and (d) %CVC_max_ across experimental conditions. Repeated measures ANOVAs were used to compare change in (a) TRG and GLU before and after HFM, (b) %CVC_max_ before and after HFM-TT, and (c) changes in core temperature during the HFM-TT trial.

A linear regression was conducted to examine the unique influence of blood triglycerides and blood glucose on %CVC_max_ for each trial (control, HFM, or HFM-TT) and for each phase of the local heating response (initial peak and plateau). Pearson correlations denote unadjusted relationships between blood triglycerides and %CVC_max_ and between blood glucose and %CVC_max_. Standardized beta coefficients denote associations between (a) blood triglycerides and %CVC_max_ after adjusting for blood glucose and (b) blood glucose and %CVC_max_ after adjusting for blood triglycerides. Thus, the advantage of beta coefficients is their ability to show the unique contribution of each blood variable (triglycerides or glucose) to the variance of %CVC_max_.

## 3. Results

### 3.1. Blood Variables

Blood triglyceride and glucose data for all protocols are shown in Table 1. Despite the high variability, all blood triglyceride data were normally distributed as determined from the Shapiro-Wilk test. There were no differences in baseline blood triglycerides between trials (control: 84 ± 9 mg/dL; HFM: 88 ± 6 mg/dL; HFM-TT: 91 ± 9 mg/dL; *P* = 1.000 for all conditions). There was a significant increase in blood triglycerides two hours after the high fat meal compared to baseline for both the HFM trial (178 ± 16 mg/dL, *P* = 0.003) and the HFM-TT trial (173 ± 14 mg/dL, *P* = 0.002). There were no differences between the two high fat meal trials (*P* = 1.000). Baseline blood glucose did not differ between trials (control: 83 ± 5 mg/dL; HFM: 84 ± 3 mg/dL; HFM-TT: 82 ± 3 mg/dL; *P* = 1.000 for all conditions). Two hours after the high fat meal, there was a significant increase in blood glucose for both the HFM (105 ± 3 mg/dL; *P* = 0.019) and the HFM-TT (102 ± 4 mg/dL; *P* = 0.010) trials; however, there was no difference in blood glucose between the two high fat meal trials (*P* = 1.000).

### 3.2. Blood Pressure and Heart Rate

Blood pressure and heart rate data for all protocols are summarized in Table 2. Although systolic blood pressure during the HFM tended to be higher compared to the control trial (*P* = 0.078), this did not reach statistical significance. There were no statistical differences in systolic blood pressure between any of the other conditions (control versus HFM-TT: *P* = 0.975; HFM versus HFM-TT: *P* = 0.511). There were no statistical differences in either diastolic blood pressure between trials (control versus HFM: *P* = 0.912; control versus HFM-TT: *P* = 1.000; HFM versus HFM-TT: *P* = 1.000) or in MAP between trials (control versus HFM: *P* = 0.300; control versus HFM-TT: *P* = 1.000; HFM versus HFM-TT: *P* = 0.868). Heart rate increased during the HFM-TT condition (88 ± 3 beats/minute) compared to both control (61 ± 2 beats/minute; *P* < 0.05) and HFM (68 ± 3 beats/minute; *P* < 0.05) conditions. Heart rate was not statistically different between control and HFM conditions (*P* = 0.817).

### 3.3. Core Temperature

Core temperature averaged 37.31 ± 0.11°C throughout the control trial and 37.27 ± 0.10°C for the duration of the HFM trial. For the HFM-TT trial, core temperature increased from a baseline of 37.36 ± 0.13°C to 37.68 ± 0.18°C during exposure to TT. There was no statistical difference in baseline core temperature between the three trials. The heat stress during the HFM-TT trial was sufficient to significantly raise core temperature to the goal of ~0.2–0.3°C above baseline (*P* = 0.042).

### 3.4. Baseline and Maximal Cutaneous Vascular Conductance

Baseline %CVC_max_ data for all three trials is shown in Table 3. There were no statistical differences between the prelocal heating baselines for any of the three trials, suggesting that changes in baseline cannot explain the observed differences in %CVC_max_ of the initial peak or plateau during the HFM and HFM-TT trials (*P* = 1.000 for all conditions). The post-high fat meal baseline during the HFM-TT trial (i.e., at the end of TT application) was significantly greater than the prehigh fat meal baseline (*P* = 0.022), indicating that TT was sufficient to approximately double skin blood flow.

Absolute maximal CVC values averaged 2.68 ± 0.38 for the control trial, 2.47 ± 0.28 for the HFM trial, and 2.63 ± 0.27 for the HFM-TT trial. There were no statistical differences in maximal CVC between trials (*P* > 0.951 for all conditions).

### 3.5. Cutaneous Vascular Conductance: Initial Peak Responses

**Figure 2:**
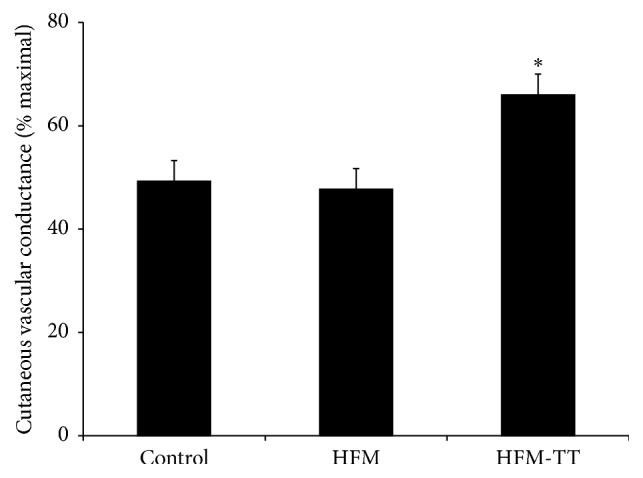
Initial peak %CVC_max_. Initial peak %CVC_max_ for the control, high fat meal (HFM), and high fat meal plus TT (HFM-TT) trials. There was no effect of the HFM on the initial peak compared to the control trial. Initial peak was augmented in the HFM-TT trial compared to the control and HFM trials. ^*∗*^ *P* < 0.05 versus control and HFM trials. Data are group mean ± SEM.

The group data for the initial peak responses are shown in Figure 2. Initial peak during the control trial averaged 49 ± 4 %CVC_max_. The initial peak during the HFM trial (48 ± 4 %CVC_max_) was not different compared to the control trial (*P* = 1.000). The initial peak during the HFM-TT trial (66 ± 4 %CVC_max_) was augmented compared to both the control (*P* = 0.002) and the HFM trials (*P* = 0.011).

### 3.6. Cutaneous Vascular Conductance: Plateau Responses

**Figure 3:**
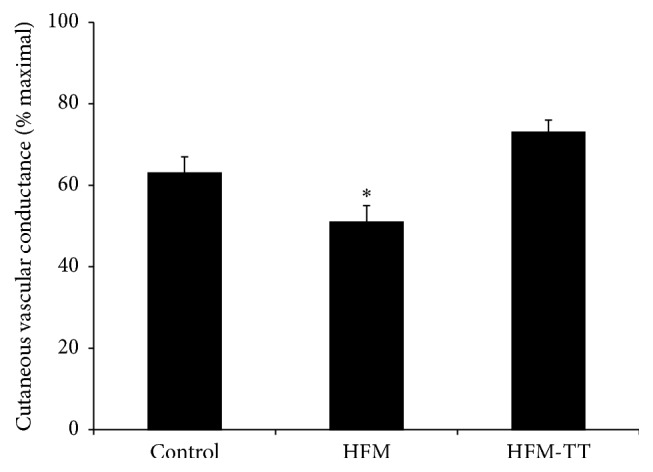
Plateau %CVC_max_. Plateau %CVC_max_ for the control, high fat meal (HFM), and high fat meal plus TT (HFM-TT) trials. Plateau in the HFM trial was attenuated compared to both the control and HFM-TT trials. There was no difference between the control and HFM-TT trials. ^*∗*^ *P* < 0.05 versus control and HFM-TT trials. Data are group mean ± SEM.

The group data for the plateau phase of the local heating response are shown in Figure 3. The %CVC_max_ values were significantly reduced in the HFM trial (51 ± 5 %CVC_max_) compared to the control (63 ± 5 %CVC_max_; *P* < 0.001) and the HFM-TT (73 ± 3 %CVC_max_; *P* < 0.001) trial. There was no statistical difference between the control and HRM-TT trials (*P* = 0.156).

### 3.7. Linear Regression Analysis of %CVC_max_ with Blood Triglyceride and Blood Glucose

Linear regression results for the initial peak are shown in Table 4. Pearson correlations and standardized beta coefficients assessing the association between (a) the initial peak %CVC_max_ and blood triglycerides and (b) the initial peak and blood glucose for any of the experimental trials (control, HFM, or HFM-TT) were not statistically significant.

Linear regression results for the plateau are shown in Table 4. In the control trial, there were no statistically significant Pearson correlations or standardized beta coefficients. In the HFM trial, there was a statistically significant unadjusted negative association between plateau %CVC_max_ and blood triglycerides (Pearson correlation is −0.827; *P* = 0.006). This association remained statistically significant after adjusting for blood glucose (standardized *β* is −0.879; *P* = 0.011). There was no statistically significant association between %CVC_max_ and blood glucose in the HFM trial. In the HFM-TT trial, there was a statistically significant unadjusted negative association between plateau %CVC_max_ and blood triglycerides (Pearson correlation is −0.739; *P* = 0.018); however, after adjusting for blood glucose, this association was no longer statistically significant (standardized *β* is −0.637; *P* = 0.064). There was no statistically significant association between %CVC_max_ and blood glucose in the HFM-TT trial.

## 4. Discussion

Data from the present study demonstrates that an acute high fat meal, and the resulting increase in blood triglycerides, negatively affects the plateau phase of cutaneous thermal hyperemia in young healthy participants. Our data further demonstrate that TT can mitigate the negative effects of a high fat meal. This beneficial effect lasts for at least one hour after cessation of the heat stimulus.

### 4.1. Initial Peak: Sensory Nerve-Mediated Cutaneous Vasodilation

The cutaneous vasodilation in response to local heating is biphasic, where cutaneous sensory nerves and TRPV-1 channels largely mediate the initial rapid vasodilation, with a modest contribution of NO (~25%). Data from the present study suggest that an acute high fat meal has no effect on the sensory nerve-mediated cutaneous vasodilation and that increased blood triglycerides do not affect sensory nerve function in young healthy humans (Figure 2). Regression analyses providing unadjusted and adjusted associations further suggests that the initial peak %CVC_max_ is not significantly correlated with either blood triglycerides or blood glucose in either of the high fat meal trials (Table 4).

As shown in Figure 2, the initial peak in the HFM-TT protocol was significantly augmented compared to both the control and high fat meal only trials. These data collectively suggest that an acute high fat meal does not negatively affect sensory nerve function and that TT can improve sensory nerve-dependent vasodilation even in young, healthy humans. Previous studies have suggested that a high fat meal and increased triglycerides can negatively affect sensory nerve function and may serve as an early risk factor for diabetic neuropathy [52–54]. It is unclear why we did not observe a detrimental effect of a high fat meal on sensory nerve-dependent vasodilation; however, these previous studies have been performed either in humans with type 2 diabetes or in Zucker diabetic fatty rats, which may suggest that chronic elevations in triglycerides can have detrimental effects on sensory nerve function. It is possible that the acute elevation of blood triglycerides in our study was not of sufficient duration to affect cutaneous sensory nerve function. Our data indicating that TT can augment cutaneous sensory nerve function in young, healthy humans is salient in that it suggests that this therapy may be a means to help prevent, or slow the onset of, diabetic neuropathy in individuals most at risk, such as those with prediabetes, obesity, or increased triglycerides.

### 4.2. Plateau: Nitric Oxide-Mediated Cutaneous Vasodilation

The prolonged secondary rise in CVC to a plateau during local heating is largely mediated by NO. We utilized a new local heating protocol developed by Choi et al. who showed that local heating of the skin to 39°C, rather than the traditional 42°C, resulted in robust, yet submaximal, cutaneous vasodilation, with a plateau phase containing a substantial (~80%) NO component [34]. The present study revealed that a single high fat meal significantly attenuates the plateau phase of cutaneous thermal hyperemia (Figure 3), indicating that an acute high fat meal and elevated blood triglycerides negatively affect cutaneous endothelial-dependent vasodilation.

Consumption of a high fat meal resulted in an increase in both blood triglycerides and blood glucose under both the thermoneutral and TT conditions; the attenuated plateau CVC could thus be due to either the increase in blood triglycerides or blood glucose or both. Plateau CVC in both the high fat meal and TT trials showed a strong negative correlation with blood triglycerides while there was no significant correlation with blood glucose (Table 4). Based on the standardized beta coefficients from the regression analysis, it appears that the decrement in plateau %CVC_max_ following consumption of a high fat meal is associated with the increase in blood triglycerides rather than the increase in blood glucose. It is important to note that prospective studies and data are required to determine a causative role for blood triglycerides influencing microvascular function. Nevertheless, standardized beta coefficients in this study suggest that, for every one standard deviation increase in blood triglycerides, there is a 0.879 standard deviation decrease in %CVC_max_, after controlling for blood glucose. High levels of circulating triglycerides immediately after a high fat meal have a strong, detrimental effect on cutaneous microvascular vasodilation. Inasmuch as the plateau phase is ~70–80% NO-dependent, this indicates that increased triglycerides following a high fat meal negatively affect endothelial NO-dependent vasodilation. It is of interest that there was no significant correlation between plateau CVC and blood triglycerides or blood glucose during the control trial, suggesting that basal/fasting blood triglycerides and blood glucose do not affect cutaneous microvascular responses; however, there is a strong negative correlation following consumption of a high fat meal that significantly increases blood triglycerides. Although the decrement in cutaneous microvascular function following a high fat meal is associated with blood triglycerides, we cannot exclude the possibility of more complex interactions occurring at the second messenger level. For example, protein kinase C theta activation elicits vasoconstriction and has been shown to be associated with obesity and insulin resistance; the protein kinase C theta-mediated vasoconstriction occurs through a complex series of events, including Akt inhibition and ERK1/2 [55]. Whether similar complex interactions affect microvascular endothelial function in response to a high fat meal requires further investigation.

It is possible that TT improved cutaneous microvascular endothelial function following a high fat meal either by increasing bioavailable NO secondary to reduced oxidative stress or by directly increasing NO via a shear stress mechanism. High fat meals have been shown to increase oxidative stress [22, 23, 25, 56] where inflammation associated with a high fat meal causes triglyceride-rich lipoproteins to accumulate in the blood. In humans, impaired flow-mediated dilation, a measure of conduit artery function and, presumably, NO-dependent vasodilation, associated with serum triglycerides has been shown to be reversed by administration of vitamins C and E [57–59]; antioxidant properties of vitamins C and E appear to counteract oxidative stress induced by a high fat meal [57–60]. Human studies have further demonstrated an increase in exhaled nitric oxide, an indicator of airway inflammation, and reduced pulmonary function following a high fat meal [27, 30]. In the human skin microvasculature, antioxidant treatment was shown to mitigate the detrimental effects of a high fat meal [23, 25]. The augmented plateau CVC responses in the TT trial of the present study may therefore be the result of reduced oxidative stress, which restores sensory nerve function and results in reduced bioavailable NO. Studies in both humans and animals have shown that sauna therapy can improve endothelial function, increase bioavailable NO, and reduce oxidative stress and inflammation [43, 61].

Exposure to TT may also improve cutaneous vascular responses following a high fat meal by directly increasing NO production. Increased skin blood flow is a direct result of increased core temperature and the resultant shear stress from increased blood flow through the cutaneous microvasculature may act as a stimulus for additional NO bioavailability [61, 62]. Indeed, sauna therapy has been shown to improve endothelial function in patients with risk factors for coronary heart disease and in chronic heart failure [45, 63]. Whether shear stress is an important stimulus for NO in the cutaneous microvasculature remains equivocal as reactive hyperemia, a stimulus known to significantly elevate shear stress, does not appear to be an NO-dependent response in the cutaneous microvasculature of humans [64, 65]. It is thus possible that the improvements in microvascular function observed in this study following TT are independent of a shear stress mechanism.

Regression analysis further suggests that TT improves both initial peak and plateau CVC independent of decreases in either blood triglycerides or blood glucose. In both of the high fat meal trials, blood triglycerides peaked approximately 60 minutes after the high fat meal and blood glucose peaked approximately 30 minutes after the high fat meal and there was no difference in the magnitude of blood triglycerides or blood glucose between control and TT conditions. Exposure to TT augmented both initial peak and plateau CVC independent of alterations (i.e., reductions) in either blood triglycerides or blood glucose. Based on these observations, the beneficial effects of TT appear to occur at the local neurovascular level rather than by affecting metabolism (production and/or clearance) of triglycerides and glucose. It is also possible the augmented CVC during the TT trial could have been driven by elevations in baseline following the mild heat stress protocol; however, baseline values just prior to the local heating protocol were not different than baseline in either the control or high fat meal only protocol, which would argue against an elevation in baseline for the augmented CVC responses in the TT protocol (Table 3).

### 4.3. Participant Demographics

Data from previous studies suggest that race/ethnicity can affect the vascular response to a high fat meal [23, 25, 47, 48]. Previous studies have also shown that vascular function is greatly compromised in Asians compared to Caucasians following a high fat meal [23, 25, 47, 48]. Yet, Asians have a more pronounced response to antioxidants than Caucasians. Caucasians also appear to have a normal thermal hyperemic response following a high fat meal [47–49]. In the present study, we found that a high fat meal compromises the thermal hyperemic response in a predominantly Caucasian (*n* = 6, 75%) participant population. The differences in findings between previous studies and the present study are most likely due to methodological differences. The previous studies heated the skin for ~6 minutes, whereas our local heating protocol lasted ~30 minutes.

In this study, we chose to investigate the effect of a single high fat meal and TT on young, apparently healthy participants. To our knowledge, there are no reports on the effect of a high fat meal on both sensory nerve- and NO-dependent cutaneous vasodilation simultaneously. There are also no reports regarding the combined effects of a high fat meal and TT on cutaneous neurovascular function in young, healthy participants. Pathologies such as diabetes and cardiovascular disease are known to attenuate both sensory nerve- and NO-dependent cutaneous vasodilation. To avoid potential confounding influences of pathology on cutaneous vasodilation, we chose to utilize a young, healthy population. Whether the observations from the present study translate to individuals with cardiometabolic diseases remains to be determined.

## 5. Limitations

Rapid, nonpainful local skin heating has been widely used as a noninvasive means to assess microvascular endothelial function in humans. While we based our protocol on a previously established and tested heating protocol, we did not specifically block sensory nerves or inhibit the production of NO in this study and therefore we cannot be certain that our results are entirely reflective of reductions in sensory nerve-dependent vasodilation or endothelial NO-dependent vasodilation. In order to directly assess the effect of a high fat meal and circulating triglycerides, sensory nerve block via EMLA cream and intradermal infusion of L-NAME, a nonspecific NOS inhibitor, via microdialysis, would need to be performed. Intradermal microdialysis is minimally invasive, but it may not be a feasible means to assess microvascular endothelial function in clinical settings; a relatively simple, noninvasive test, such as nonpainful local heating, can be easily implemented in clinical settings to assess both sensory nerve and endothelial function in a wide range of populations. Despite this limitation, our data demonstrate that a single high fat meal can indeed blunt microvascular vasodilation and we are confident that using the local heating protocol developed by Choi et al. [34] represents a noninvasive means by which to assess microvascular function in humans. A second limitation to the present study is the small sample size. It is possible that some of the associations assessed by linear regression did not reach statistical significance due to the small sample size.

## 6. Conclusion

We found that a single high fat meal attenuated both initial peak and plateau CVC responses to local heating compared to control while exposure to TT prior to, during, and after the consumption of a high fat meal restored cutaneous vascular responses. A novel aspect of this study is the use of local heating to simultaneously assess sensory nerve function and endothelial function in response to a high fat meal. These data indicate that an acute high fat meal negatively affects microvascular endothelial function while the application of TT can restore microvascular endothelial function.

## Figures and Tables

**Table 1:** Blood triglyceride and blood glucose data. Both blood triglycerides and blood glucose increased above baseline in both high fat meal trials. HFM: high fat meal; HFM-TT: high fat meal plus thermotherapy. ^*∗*^ *P* < 0.0125 versus baseline within the same trial. Values are means ± SEM.

	Blood triglycerides (mg/dL)		Blood glucose (mg/dL)	
	Baseline	After high fat meal	Baseline	After high fat meal
Control	84 ± 9		83 ± 5	
HFM	88 ± 6	178 ± 16^*∗*^	84 ± 3	105 ± 3^*∗*^
HFM-TT	91 ± 9	173 ± 14^*∗*^	82 ± 3	102 ± 4^*∗*^

**Table 2:** Blood pressure and heart rate data. There were no differences in any of the blood pressure values between the three trials. HFM: high fat meal trial; HFM-TT: high fat meal plus thermotherapy trial. Values are means ± SEM. ^*∗*^ *P* < 0.05 versus control and HFM conditions.

	Systolic blood pressure (mmHg)	Diastolic blood pressure (mmHg)	Mean arterial blood pressure (mmHg)	Heart rate (beats/minute)
Control	116 ± 5	76 ± 5	89 ± 5	61 ± 2
HFM	120 ± 5	77 ± 5	91 ± 5	68 ± 3
HFM-TT	118 ± 6	75 ± 4	90 ± 4	88 ± 3^*∗*^

**Table 3:** Baseline %CVC_max⁡_ data. Baseline values for the three experimental trials. Pre-HFM was defined as the three-minute period just prior to either the thermoneutral or TT; post-HFM was defined as the three-minute period immediately preceding the end of either the thermoneutral or TT; pre-local heating was defined as the three-minute period just prior to beginning the local heating protocol. HFM: high fat meal; HFM-TT: high fat meal plus mild heat stress trial. ^*∗*^ *P* < 0.05 versus same time point compared to HFM; ^#^ *P* < 0.05 versus pre-HFM and pre-local heating within the same trial. Values are means ± SEM.

	Control trial	HFM trial	HFM-TT trial
Pre-HFM		14 ± 2	18 ± 3
Post-HFM		13 ± 2	32 ± 3^*∗*#^
Pre-local heating	17 ± 3	17 ± 3	21 ± 5

**Table 4:** Linear regression for the correlation between %CVC_max⁡_ and blood triglycerides and blood glucose for both the initial peak and plateau phase of cutaneous thermal hyperemia. HFM: high fat meal; HFM-TT: high fat meal + thermotherapy; TRG: blood triglycerides; GLU: blood glucose. *∗* indicates statistically significant Pearson correlation and standardized beta coefficient.

	Control		HFM		HFM-TT	
	TRG	GLU	TRG	GLU	TRG	GLU
*Initial peak*						
Pearson correlation	0.424	0.252	0.267	−0.145	−0.268	−0.433
*P* value	0.148	0.273	0.262	0.366	0.260	0.142

Standardized *β* coefficient	0.434	0.269	0.305	−0.203	−0.424	−0.552
*P* value	0.313	0.518	0.509	0.657	0.309	0.201

*Plateau*						
Pearson correlation	0.542	0.162	−0.827	0.108	−0.739	0.540
*P* value	0.082	0.351	0.006^*∗*^	0.399	0.018^*∗*^	0.083

Standardized *β* coefficient	0.550	0.183	−0.879	0.275	−0.637	0.361
*P* value	0.195	0.639	0.01^*∗*^	0.275	0.064	0.238
